# Polaronic Contributions to Friction in a Manganite Thin Film

**DOI:** 10.1002/advs.202003524

**Published:** 2021-02-18

**Authors:** Niklas A. Weber, Hendrik Schmidt, Tim Sievert, Christian Jooss, Friedrich Güthoff, Vasily Moshneaga, Konrad Samwer, Matthias Krüger, Cynthia A. Volkert

**Affiliations:** ^1^ Institute of Materials Physics University of Göttingen Göttingen 37077 Germany; ^2^ The International Center for Advanced Studies of Energy Conversion (ICASEC) University of Göttingen Göttingen 37077 Germany; ^3^ Institute of Physical Chemistry University of Göttingen Göttingen 37077 Germany; ^4^ 1st Physics Institute University of Göttingen Göttingen 37077 Germany; ^5^ Institute for Theoretical Physics University of Göttingen Göttingen 37077 Germany

**Keywords:** atomic force microscopy, friction, friction force microscopy, manganite, polarons

## Abstract

Despite the huge importance of friction in regulating movement in all natural and technological processes, the mechanisms underlying dissipation at a sliding contact are still a matter of debate. Attempts to explain the dependence of measured frictional losses at nanoscale contacts on the electronic degrees of freedom of the surrounding materials have so far been controversial. Here, it is proposed that friction can be explained by considering the damping of stick‐slip pulses in a sliding contact. Based on friction force microscopy studies of La_(1−_ *_x_*_)_Sr*_x_*MnO_3_ films at the ferromagnetic‐metallic to a paramagnetic‐polaronic conductor phase transition, it is confirmed that the sliding contact generates thermally‐activated slip pulses in the nanoscale contact, and argued that these are damped by direct coupling into the phonon bath. Electron‐phonon coupling leads to the formation of Jahn–Teller polarons and to a clear increase in friction in the high‐temperature phase. There is neither evidence for direct electronic drag on the atomic force microscope tip nor any indication of contributions from electrostatic forces. This intuitive scenario, that friction is governed by the damping of surface vibrational excitations, provides a basis for reconciling controversies in literature studies as well as suggesting possible tactics for controlling friction.

## Graphical abstract

Friction can be controlled at a manganite surface using the phase transition between the metallic and polaron conductor phases. The sliding tip generates stick‐slip pulses in the contact interface, which are more effectively damped in the polaron conductor phase due to strong electron‐phonon coupling.

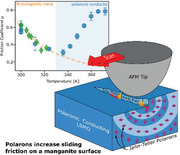

## 1 Introduction

Understanding and controlling friction is a long‐standing, major topic in both research and application. The earliest approaches to controlling friction have focused on changing the sliding contact by modifying surface roughness or adding lubricants. More recently, atomic force microscope (AFM) methods have allowed basic studies of friction which intensively address how energy is dissipated at a sliding contact.^[^ ^1^, ^2^, ^3^, ^4^ ^]^ In addition to the atomic interactions in the contact interface, it has been found that phonon dissipation mechanisms,^[^ ^5^ ^]^ conduction electron excitation and drag,^[^ ^6^ ^]^ and electrostatic forces^[^ ^7^, ^8^, ^9^ ^]^ in the surrounding materials can significantly influence the friction between two bodies. This offers the intriguing possibility to change friction by varying material properties, without having to control the exact nature of the contact interface, which is strongly affected by operating conditions. Eventually, the goal is to bridge from the nanoscale contacts of AFM‐based studies to macroscale contacts, which are composed of many interacting nanoscale contacts, with the hope of developing a physical basis for optimizing engineering friction.

Although our understanding of the basic mechanisms of energy dissipation at a sliding contact is surprisingly vague, there are two remarkable characteristics of friction which we now understand. The first is the century‐old observation that the friction force *F*_f_ on a macroscopic object is proportional to the normal force *F*_N_ between the object and the surface on which it moves and not to the macroscopic contact area.^[^ ^10^ ^]^ This at‐first confusing observation seems to contradict the basics of classical mechanics, but was finally resolved by the understanding that the true contact area *A* between two objects is composed of many nano‐asperities, and is much smaller than the apparent contact area; the true contact also increases roughly linearly with the normal force.^[^ ^10^, ^11^ ^]^ Since then, a number of studies have indeed confirmed that the friction forces at both single asperity nano‐contacts and multi‐asperity macro‐contacts scale with the true contact area,^[^ ^12^, ^13^, ^14^, ^15^, ^16^ ^]^ so that

(1) $$F_{f}=\;\tau A$$

where *τ* is a shear stress which is required to generate and propagate the excitations that allow sliding in the contact area.^[^ ^17^ ^]^ It is conceptually analogous to the Peierls stress required for dislocation glide^[^ ^18^, ^19^ ^]^ but is expected to be smaller due to slightly increased interatomic distances due to incommensurability, defects, and the presence of (often amorphous) oxide and contamination layers.

The last several decades have shown great progress in using AFM to identify the excitations that are stimulated at nanoscale sliding contacts. It is widely observed in both experiment and theory that sliding contact does not proceed continuously, but by stick‐slip events.^[^ ^14^, ^20^, ^21^, ^22^, ^23^ ^]^ The stick‐slip events are observed at all length scales, accounting for earthquakes^[^ ^24^ ^]^ and atomic‐scale instabilities,^[^ ^20^ ^]^ as well as the acoustics of violins, squeaky doors, grasshoppers, and squealing brakes. With the exception of contacts containing only a few molecules, interface sliding should be envisioned as the motion of slip pulses through the contact interface, where the local slip velocity far exceeds the average slip velocity of the contact.^[^ ^19^ ^]^ Even cases of apparent continuous sliding are often attributed to local slip pulses moving through the interface.^[^ ^25^ ^]^ Thus, the average AFM tip or scan velocity can be expressed as

(2) $$v\;=\;A\rho v_{\mathrm{sp}}$$

where *A* is the contact area, *ρ* is the areal density of active slip pulses (so that *Aρ* is the average number of active slip pulses in the contact), and *v*_SP_ is their average slip pulse velocity. This equation is analogous to Orowan's equation for the strain rate due to dislocation glide. A single slip pulse should be viewed as a localized slip wave that moves through the interface with an average slip pulse velocity *v*_sp_ and, once it traverses the contact area, causes a discrete sliding displacement of the tip. The forward slip distance of a slip pulse *b* can vary from meters for earthquakes to Angstroms for dislocations. In the case of a general incommensurate or disordered nanoscale contact, *b* is not conserved (in contrast to the case for dislocation Burgers vectors) and will vary from position to position within the contact by converting the slip displacement to local elastic strains. The AFM tip displacement due to slip pulse traversal will equal the average value of *b* along the contact area perimeter, and is thus not fixed at an interatomic distance or lattice spacing (or a multiple thereof). We note that not all 3D Molecular Dynamics simulations of atomic‐scale sliding friction show evidence of slip pulse generation, which may be because of the high speeds often used in such studies.^[^ ^26^ ^]^

Stick‐slip behavior at the atomic scale,^[^ ^27^, ^28^, ^29^ ^]^ through the mesoscale,^[^ ^22^ ^]^ to the macroscale^[^ ^23^ ^]^ has been successfully modeled using minimal 1D models such as the Prandtl–Tomlinson and Frenkel–Kontorova models. The remarkable success of these models lies in the fact that they can account for the strongly nonlinear dependence of friction on scan velocity, which is the second remarkable characteristic of friction. By describing the motion of an AFM tip as spring‐loaded thermally‐activated motion over an energy barrier, a quantitative agreement has been obtained with scan velocity dependences, provided the ratio of barrier height to spring stiffness is high enough that an instability occurs.^[^ ^28^, ^29^, ^30^, ^31^, ^32^, ^33^, ^34^ ^]^ In essence, the models are based on an Arrhenius‐type law for the 1D average slip pulse velocity of the form^[^ ^30^, ^31^ ^]^

(3) $$v_{\mathrm{sp}}=\;\omega_{o}L\exp\left(\frac{-\Delta E}{kT}\right)$$

where *ω*_0_ is an attempt frequency, *L* is a factor with units of length that depends on the contact stiffness of the experimental set‐up and temperature,^[^ ^30^, ^31^, ^32^ ^]^ and Δ*Ε* is the activation energy barrier which must be overcome for a slip to occur. The basic assumptions that lead to this equation are that a slip event is critically damped, meaning that neither reverse slip jumps (overdamped) nor multiple jumps from inertial effects (underdamped) occur. There is evidence from atomic stick‐slip behavior to support this idea,^[^ ^27^ ^]^ and near‐critical behavior may, in fact, be an emergent property in sliding contact.^[^ ^25^, ^35^ ^]^

Assuming that the activation barrier for slip Δ*Ε* is reduced from the unloaded value *Ε*_0_ of the activation barrier by the force *F*_SP_ driving the slip event,^[^ ^30^, ^31^, ^32^ ^]^ an equation for the friction force can be obtained that captures the basic features of the scan velocity dependence, namely that the friction force is finite in the limit of zero scan velocity and shows a weak dependence on scan velocity. A number of different approximations and validity ranges have been considered,^[^ ^26^, ^28^, ^29^, ^30^, ^31^, ^32^, ^33^, ^34^ ^]^ all leading to a logarithmic dependence of the friction force on the scan velocity. Using a linear approximation Δ*Ε = Ε*_0_−*bF*_SP_ *π^−1^* to describe the dependence of the activation barrier on the force acting on an individual slip pulse *F*_SP_,^[^ ^30^, ^32^ ^]^ one obtains an equation that can be used to approximately describe the dependence of the friction force on scan velocity,

(4) $$F_{f}=F_{\mathrm{ph}}\;=\frac{\pi A}{b^{2}w}\;\left[E_{o}+kT\ln\left(\frac{v}{v_{o}}\right)\right]$$

In obtaining Equation (4), we have used Equation (1) to Equation (3), set *v*_0_ *= Aρω*_0_ *L*, and related *F*_SP_ to the shear stress *τ* in the contact interface as *F*_sp_ = *τbw* where *b* is the mean forward slip distance of the slip pulse. This quasi‐1D model describes the average tip velocity as a result of slip pulses that span the entire width *w* of the contact. Note that the energy barrier *E*_0_ and shear stress *τ* increase with the normal force,^[^ ^31^ ^]^ analogous to the dependence of dislocation glide on normal force, so that the friction force *F*_f_ depends on the normal force *F*_N_ both through the contact area *A* and *E*_0_.

Equations for thermally‐activated stick‐slip behavior with forms similar to Equation (4) have been derived from the Prandtl–Tomlinson model and widely used in the literature to discuss the scan velocity dependence of friction.^[^ ^28^, ^30^, ^31^, ^33^, ^34^ ^]^ The models are able to fit the data quite well, but surprisingly, predict that friction should only depend on surface structure (through *E*_0_, *b*, and *ρ*) and contact area (*A*), and not on the inherent dissipation rates in the surrounding materials.^[^ ^27^ ^]^ In reality, it is clear that thermally activated jumps at surfaces couple directly to the macroscopic degrees of freedom of the underlying materials, as has been widely discussed within the context of adsorbate vibration relaxation.^[^ ^36^ ^]^ The predicted independence from inherent dissipation within the Prandtl–Tomlinson model follows from the assumption of critical damping, which is supported by both experiment and simulation at the atomic scale.^[^ ^27^, ^35^ ^]^ In fact, thorough modeling of friction for a wide range of dissipation rates show that the friction force increases with the dissipation rate in both the underdamped and overdamped regimes, but is relatively constant in the critically‐damped regime,^[^ ^29^ ^]^ in good agreement with Kramers reaction rate theory.^[^ ^37^ ^]^ The question of which regime best describes experimental data remains open.

In fact, a number of experimental studies show a clear dependence of friction on the surrounding material properties,^[^ ^6^, ^7^, ^8^, ^9^, ^38^, ^39^, ^40^, ^41^, ^42^, ^43^ ^]^ suggesting that it is time to move beyond the widely used critically‐damped Prandtl–Tomlinson model (e.g., Equation (3) and Equation (4)), despite its impressive success accounting for the scan velocity dependence of friction. The most compelling experimental studies for the effect of surrounding material properties have investigated friction across the superconducting transition. Despite relatively unchanged surface bonding and structure, a clear increase in friction on transitioning from the superconducting to the normal state has been observed in a variety of materials.^[^ ^6^, ^41^, ^42^, ^43^ ^]^ One explanation given for the behavior is based on viscous damping of the tip by electronic excitations: in the superconducting state, the electrons form Cooper pairs that exhibit an energy gap in their excitation spectrum, while in the normal state quasi‐free electrons are easily excited and dissipate the sliding energy. An electronic friction force *F*_el_ is simply added to the total friction force as *F*_f_ = *F*_ph_ *+ F*_el_, where the phononic friction *F*_ph_ results from the slip pulse excitations described above in Equation (4). The electronic friction force *F*_el_ *= −B*_el_ *v* is assumed to represent damping by normal carriers near the Fermi energy through a viscous damping coefficient *B*_el_ as a result of electromagnetic or van der Waals interactions with the tip.^[^ ^6^, ^41^, ^43^ ^]^ Although van der Waals interaction forces are believed to make significant contributions to the adhesion force,^[^ ^44^ ^]^ theoretical values for the van der Waals friction based on generating electronic excitations near the Fermi energy and then dragging them behind the tip^[^ ^1^ ^]^ (Coulomb drag) deliver forces that are much too small (on the order of 10^−18^ N for typical AFM measurement conditions) to account for the measured changes in the sliding friction force on the order of 10^−10^ N or larger.^[^ ^41^, ^43^ ^]^ On the other hand, there is good agreement with the much smaller forces measured in non‐contact friction studies,^[^ ^6^ ^]^ where presumably the slip pulse excitations described in Equation (4) are not active. This suggests that viscous electronic damping of van der Waals interactions with the tip is active and presumably contributes to sliding contact friction; it is simply completely overshadowed by the energy dissipated in the slip pulse excitations. This same problem with the order of magnitude of possible contributions from electronic excitations has been encountered when trying to explain the effect of doping and carrier density on the sliding friction of various semiconductors,^[^ ^7^, ^40^ ^]^ although it is conceivable that the local electric fields generated in these studies might be large enough to modify the near‐surface electronic properties.

Since the energies required to generate electronic excitations through electrodynamic interactions with the sliding tip or to drag image charges (Coulomb drag) are both too small to explain the observed correlation between material properties and contact sliding friction, the effect of electrostatic forces has also been considered.^[^ ^7^, ^38^, ^40^, ^41^, ^43^ ^]^ Forces both parallel and perpendicular to the surface normal can be generated depending on the distribution of charges or dipoles in the contacting materials. Electrostatic normal forces have the effect of increasing *A* in Equation (1) described by elastic contact mechanics (e.g., Hertz contact theory), while net electrostatic forces parallel to the sample surface will directly add to the friction forces. In some cases, an order‐of‐magnitude agreement with the experiment has been achieved, but quite large and specific trapped charge densities have often been required to explain the measurements. For example, in order for electrostatic forces to contribute to the lateral forces felt by the tip, the “line of charges” left in the wake of the sliding tip must have lifetimes long enough to slow the advancing tip but short enough to have annihilated by the time the tip is rescanned along the same path.^[^ ^7^, ^38^ ^]^ Such highly specific scenarios have not yet been verified, although they would offer promising methods to control friction by tailoring electric fields.^[^ ^45^ ^]^

In this paper, we investigate nanoscale single asperity sliding friction at the surface of La_(1−_ *_x_*_)_Sr*_x_*MnO_3_ (LSMO) films (*x =* 0.2 and 0.3) while heating through transitions from the ferromagnetic (FM) metal to a paramagnetic polaronic conductor (PM) state. The transitions in these films allow us to probe the effect of electronic and phononic degrees of freedom on friction without a change in the crystal structure. We use experimental conditions where the contact contains many atoms (ca. 100), rather than attempting single‐atom contacts, in order to probe the behavior of typical nano‐asperities that make up macroscopic contacts. We observe a clear increase in friction on crossing from the metallic to small polaron hopping conductivity state, which is due to the damping of the slip pulse excitations generated in the sliding contact. We argue that the strong increase in electron‐phonon coupling at the transition causes a change in the lifetime of the slip pulses and can quantitatively account for the excess friction in the polaronic phase. This explanation is distinct from many previous explanations, which were based on van der Waals and Ohmic losses and were unable to quantitatively account for the observed friction.

## 2 Results and Discussion

### 2.1 Friction and Adhesion Forces at the Phase Transition

LSMO films with *x =* 0.2 and *x =* 0.3 were chosen for the friction studies because they manifest temperature‐driven transitions in electrical (*T*_MM_) and magnetic (*T*_C_) properties without changes in the bulk film crystal structure and in atomic bonding. At low temperatures, the films are FM with metallic conduction governed by the double exchange mechanism. They become PM above the transition temperatures with strongly increased electrical resistivity due to the transition to a small polaron conductor. The strong increase in electron‐phonon coupling results in the localization of charge carriers at the Jahn–Teller distortions to form small polarons.^[^ ^46^, ^47^, ^48^, ^49^, ^50^ ^]^ We note that the transition temperatures are dependent on the exact composition (including possible oxygen vacancies) and on stresses that result from the epitaxial relation with the substrate^[^ ^51^, ^52^ ^]^ and may be different at the surface of the films due to an observed reconstruction.^[^ ^53^ ^]^ Often the two transitions overlap due to the coupling between spin, electron, and phonon degrees of freedom, but can also be shifted from each other.^[^ ^46^, ^47^, ^48^, ^49^, ^50^ ^]^ In addition to allowing systematic tests of the effects of electronic, magnetic, and phononic degrees of freedom on friction, the manganite films offer potential technological application since it has recently been shown that resistive switching of manganite films offers a practical tactic for controlling friction without having to vary the temperature.^[^ ^54^ ^]^

A 6 nm thick film of La_0.7_Sr_0.3_MnO_3_ (*x =* 0.3 specimen) was fabricated on a buffered SrTiO_3_ substrate by metal‐organic aerosol deposition,^[^ ^55^ ^]^ with a FM‐PM transition at *T*_C_ *=* 338 K and a metal‐like to hopping small polaron conductivity transition at *T*_MM_ *=* 330 K. A 70 nm thick film of La_0.8_Sr_0.2_MnO_3_ (*x =* 0.2 specimen) was fabricated on SrTiO_3_ by sputter deposition and exhibits a FM‐PM transition at *T*_C_ *=* 220 K and a metal‐polaronic conductor transition at *T*_MM_ *=* 187 K. The crystal structure is rhombohedral both above and below the transitions ($R\bar{3}c$ space group) and strained due to the epitaxial relation with the underlying substrates.^[^ ^51^ ^]^ Measurements to determine crystal structure, film thickness, magnetic properties, and resistivity are summarized in the Supporting Information for both films (Figures S1–S10, Supporting Information).

**Figure 1:**
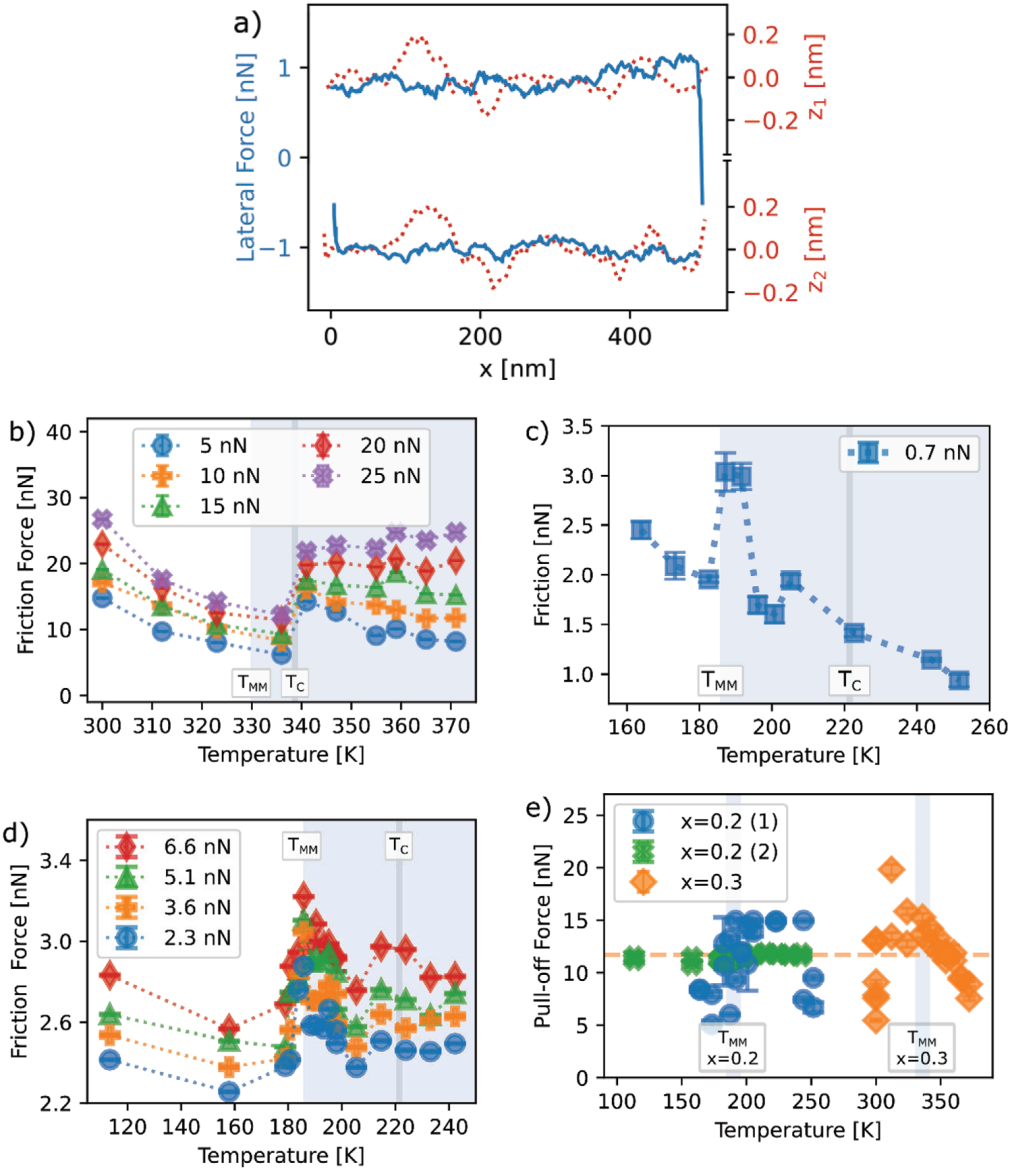
Friction and adhesion forces near the FM metal to PM polaronic conductor transition for *x =* 0.2 and *x =* 0.3 LSMO films. a) Lateral friction force loop (solid lines) and topography (dotted lines) for the *x =* 0.2 film obtained at an applied normal force of 0.7 nN and a scan velocity of 2.5 µm s^−1^ showing a strong hysteresis and stick‐slip activity. b) Average friction forces for the *x =* 0.3 film as a function of temperature for different applied normal forces at a scan velocity of 2.5 µm s^−1^. c,d) Two measurement series with different cantilevers of average friction forces for the *x =* 0.2 film as a function of temperature for different normal forces at a scan velocity of 0.25 µm s^−1^. e) Adhesion forces estimated from pull‐off force measurements for the *x =* 0.3 and *x =* 0.2 films. The error bars are given by the standard error; the number of friction scans used to determine average friction forces lies between 100 and 256 (see Experimental Section).

Nanoscale friction force measurements^[^ ^56^ ^]^ were performed on heating through the transition temperature using AFM‐based lateral force microscopy for a range of normal loads and scan speeds. The AFM studies were performed in a UHV environment in order to avoid complications from water condensation and other surface contamination. Details of the force calibration and scaling as well as data analysis are described in the Experimental Section. The normal forces *F*_N_ between the conducting nanoscale Si tip and the film were kept below 30 nN to avoid detectable wear contributions. A typical friction loop for an applied normal force of 0.7 nN is shown in **Figure** **1**a. Including the adhesion force (Figure 1e) gives an actual normal force of about 12 nN and a true contact area of about *A* = 5 nm^2^, according to elastic Hertz contact theory and using the nominal tip radius of 10 nm (see Experimental Section and Figure S11, Supporting Information). According to Equation (1), the friction force then corresponds to an interface shear stress on the order of *τ =* 2 GPa, which is reasonable, roughly a factor of 5 smaller than the Peierls barrier in perovskite oxides.^[^ ^57^ ^]^ A comparison of the friction force with topography (Figure 1a) shows that the ≈10% variations in the magnitude of the friction force during the forward and backward traces do not correlate with topography (see also the Experimental Section and Figures S12,S13, Supporting Information). The variations in friction are attributed to slip pulses traversing the contact. To first approximation in the quasi‐1D model used above, the average forward slip distance of the slip pulses can be estimated from the 10% force variations in Figure 1a as $b\;=\;10\%\cdot \;\sqrt{4A/\pi }=$ 0.25 nm,^[^ ^56^ ^]^ which is a reasonable number in that it is comparable to an interatomic distance.

Friction forces *F*_f_ were obtained for each normal load and temperature by averaging the magnitude of the forces for many friction loops (Figure 1b–d). The friction forces for both films show a gradual decrease with increasing temperature followed by an abrupt increase at the transition from the metallic to polaronic conducting state. Note that the large temperature difference (35 K) in the electrical and magnetic transition temperatures in the *x =* 0.2 film, allows us to identify that the friction change is correlated with the onset of polaron formation and not with a change in magnetic order (Figure 1c,d). In contrast, the transitions for the x = 0.3 films are too close (within 10 K) (Figure 1b) to distinguish which causes the friction change (see the Experimental Section for a discussion of specimen temperature accuracy), so that we presume it is also here caused by the polaron formation. Depending on the normal force, the friction force then either falls again for low normal forces or stays at an elevated value for larger normal forces. Friction measurements recorded first and last in the temperature series were performed at the same temperature (room temperature for *x =* 0.3 and 250 K for *x =* 0.2) and show no significant difference relative to the error bars, so we assume no or only insignificant changes in the tip geometry or surface chemistry during the friction measurements.^[^ ^56^ ^]^ Although the friction forces for the *x =* 0.2 film are smaller than for the *x =* 0.3 film (even for the same normal force), the fact that both films, with different compositions, different thicknesses, and different transition temperatures, both show the same behavior indicates that the correlation between the friction increase and the formation of small polarons is robust. We note that switching AFM tips while measuring a given specimen can easily lead to changes in the friction forces by a factor of four, presumably due to differences in the tip geometry.

The adhesive forces between the tip and LSMO films can be estimated by the pull‐off forces that were obtained from force‐distance measurements as a function of temperature. They are plotted in Figure 1e for both films and show no systematic trends with respect to the temperature or the order of measurement. In particular, values recorded at the same temperature and first and last in the temperature series give the same value of the adhesive force, confirming that there was no measurable change in the tip geometry. The adhesion energy can be estimated as 90 mJ m^−2^ using the Derjaguin, Muller, and Toporov contact model,^[^ ^58^ ^]^ which is consistent with typical interface energies measured in AFM studies.^[^ ^59^ ^]^ The lack of an abrupt change in adhesion at the transition temperature rules out the possibility that the variations in friction are caused by changes in the contact area. This is consistent with the fact that the elastic modulus changes by less than 5% at the transition temperature^[^ ^60^ ^]^ and should lead to changes in the adhesion force on the order of 1 nN or less. Furthermore, the constant adhesion force confirms that the interatomic forces, van der Waals forces, and electrostatic forces due to Coulomb and capacitive interactions do not change significantly at the phase transition. Thus, the observed changes in friction at the transitions are due to a fundamental change in the ability of the near‐surface regions of the films to dissipate energy.

### 2.2 Friction Coefficient and Normal Force Dependence

**Figure 2:**
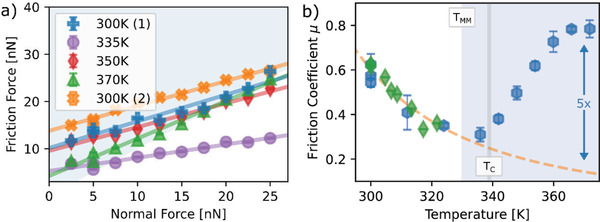
Friction of the *x =* 0.3 LSMO film. a) Friction forces show a linear dependence on the applied normal force for all temperatures both above and below the transition. b) The friction coefficient obtained from two sets of measurements with different cantilevers (blue hexagons and green diamonds) from the slopes in a) shows distinct behavior in the FM metal and PM polaronic conductor states. The dashed line shows a fit to the data with an Arrhenius dependence. The error bars are given by the standard error; the friction forces are averages of 256 measurements (see Experimental Section).

The friction force is observed to increase linearly with the normal force at each temperature, with a non‐zero intercept (**Figure** **2**a). Even in the absence of an applied normal force (*F*_N_ *=* 0), an attractive force between the tip and specimen leads to friction forces between 5 and 15 nN and to the consequence that friction forces may be larger than the normal forces (see also Figure 1a). The observed linear dependence of the friction force on the normal force is in agreement with a number of other nanoscale friction studies,^[^ ^4^, ^5^, ^9^, ^61^, ^62^ ^]^ although contact mechanical considerations for a sphere pressed against a flat surface predict sub‐linear behavior, which is also often seen.^[^ ^59^, ^63^ ^]^ The widely observed linear behavior occurs for small adhesion energies^[^ ^63^, ^64^ ^]^ and may result from deviations from a spherical tip shape, from possible dependences of *τ* on the contact area size,^[^ ^19^ ^]^ or simply from the combined effect of the normal force dependence of the contact area and the slip barrier (Equation (4)). The approximate linear dependence allows the microscopic definition of the friction coefficient (slope of the *F*_f_ vs *F*_N_ curve) to be used to characterize dissipation. The plot of the friction coefficient as a function of temperature (Figure 2b) shows two distinct trends. The friction coefficient gradually decreases with increasing temperature below the transition temperature and then abruptly increases at and above the transition temperature eventually leading to more than a doubling in value.

The gradual decrease in friction coefficient with increasing temperature is attributed to thermally activated stick‐slip behavior.^[^ ^28^, ^29^, ^33^, ^43^, ^63^, ^65^ ^]^ This decrease is often successfully fit for small friction forces with an Arrhenius dependence *F*_f_ *= F*_0_ exp(*E*_a_ */kT*), where *E*_a_ is assumed to be the rate‐limiting activation barrier for the slip event.^[^ ^5^, ^27^, ^65^, ^66^ ^]^ The best fit to the data in the FM state gives an activation energy of *E*_a_ = 0.159 ± 0.013 eV, which is similar to the activation energy values obtained in previous studies on a range of different materials.^[^ ^5^, ^38^ ^]^ Note that Equation (4) predicts a linear or power law decrease of the friction force with temperature (*v < v*_0_), which has also been widely applied in the literature.^[^ ^31^, ^34^, ^43^, ^65^ ^]^

### 2.3 Scan Velocity Dependence of Friction

**Figure 3:**
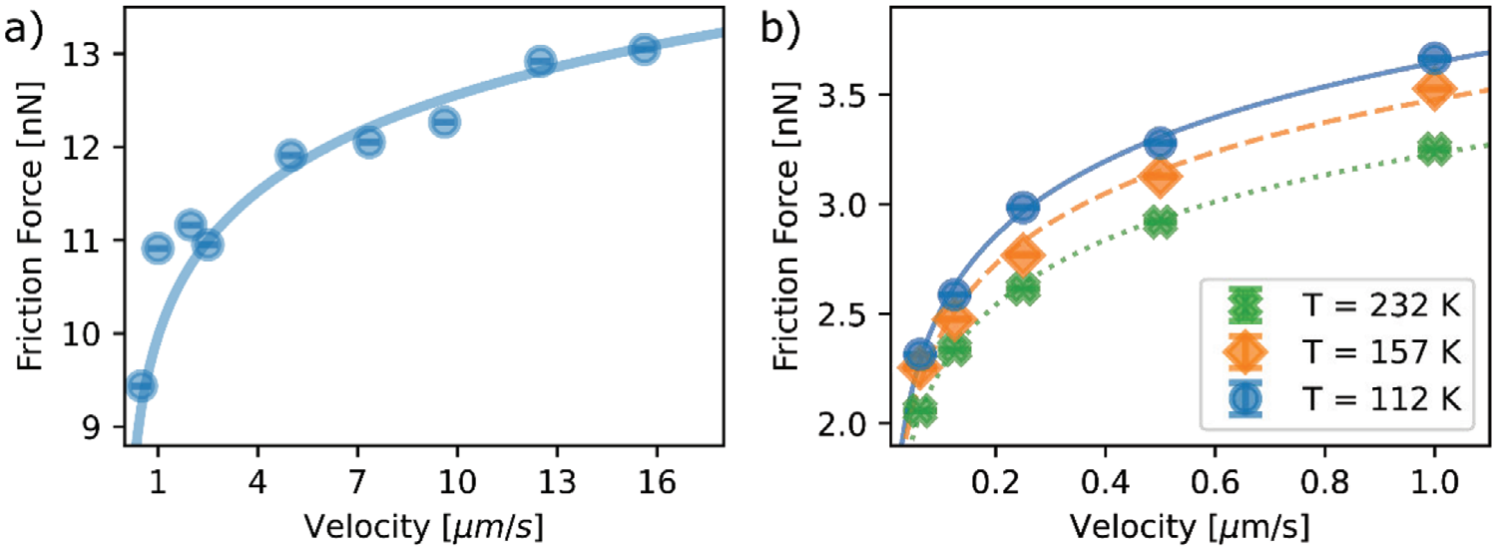
Friction forces as a function of scan velocity for the LSMO films. a) Friction force in the *x =* 0.3 film for *F*_N_ = 5 nN at room temperature in the FM state. b) Friction force in the *x =* 0.2 film for *F*_N_ = 2.3 nN below, near, and above the transition temperature. The error bars are given by the standard error; the number of friction scans used to determine average friction forces lies between 100 and 256 (see Experimental Section).

The friction forces were measured for both films as a function of scan velocity (**Figure** **3**) and show a clear non‐linear increase with velocity, both above and below the transition temperature (Figure 3b). The scan velocity dependence is well described by a logarithmic dependence, as expected from thermally activated stick‐slip models.^[^ ^30^, ^31^, ^32^, ^34^, ^65^ ^]^ Fits were made to the data using Equation (4) and setting *E*_0_ *=* 0.159 eV,^[^ ^38^ ^]^ as obtained from the temperature dependence for the x = 0.3 film. Otherwise, the fits are under‐constrained and do not stably converge. We obtain values between 1 and 3 nm s^−1^ for *v*_o_, in good agreement with the literature,^[^ ^63^ ^]^ and values for *b* which increase with the temperature from 0.04 nm at 112 K to 0.14 nm at 300 K. These values are comparable to the values estimated from Figure 1a. The idea that the slip distance might increase with temperature seems reasonable considering that the slip interface will include regions with low slip barriers which may be overcome by inertial processes.

### 2.4 Effect of Electrostatic Forces

Previous literature studies have proposed that electrostatic interactions between charges that are trapped near the specimen and tip surfaces might account for the observed dependence of friction on electronic properties.^[^ ^7^, ^38^, ^40^, ^41^, ^43^ ^]^ For instance, the increase in friction at the insulator to the metal transition of VO_2_,^[^ ^38^, ^39^ ^]^ at the superconducting to normal transition of an oxide,^[^ ^41^, ^43^ ^]^ or as a result of changes in semiconductor doping,^[^ ^7^, ^40^ ^]^ have been discussed in terms of electrostatic forces resulting from charges or fields set up by the tip‐specimen interactions. In some cases, the friction change has been accompanied by an adhesion change, lending quantitative support to the argument that electrostatic forces have increased the normal force and thereby increased the contact area.^[^ ^7^, ^38^ ^]^ In other cases, the required trapped charge densities are estimated, leading to reasonable numbers in the case of Si,^[^ ^40^ ^]^ but requiring specific time‐dependent distributions of charges in other cases.^[^ ^7^, ^38^ ^]^

**Figure 4:**
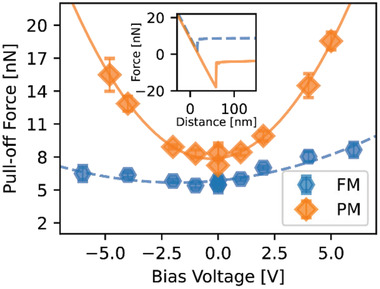
Pull‐off forces for the *x =* 0.3 film as a function of bias voltage in the FM metal (300 K) and PM polaronic conductor (380 K) states obtained from pull‐off force measurements (see inset). The adhesion has a quadratic dependence on bias in both states (solid and dashed lines). The capacitance in the PM state is roughly 5 times larger than in the FM state. The contact potential difference between the AFM tip and specimen (given by the voltage at minimum adhesion) differs by approximately 2V between the two states. The error bars are given by the standard error; the adhesion forces are each obtained from averages of 256 measurements.

In our studies, the lack of a temperature‐dependent adhesion force indicates that there are no significant differences between the Coulomb and capacitive interactions of the tip with the high and low‐temperature manganite phases under the conditions used to measure pull‐off forces. This means that contact electrification (the charges transferred between two materials when brought together and then taken apart) is not measurably different for the two phases. On the other hand, it is well‐known that the charges generated at two contacting surfaces depend on their sliding speed (tribocharging),^[^ ^67^ ^]^ so that the possibility must be explored whether tribocharging is very different in the two phases. This possibility can be indirectly ruled out by considering the tribo‐current that would be necessary above the transition to account for the measured change in friction. In Figure 1b, we observe an increase in friction force of almost 10 nN at the transition. According to Figure 2a, this requires an increase in the normal force of about 20 nN. According to measurements of the pull‐off force as a function of the applied bias voltage between the tip and the *x =* 0.3 film (**Figure** **4**), a bias voltage in excess of 6 V would be needed to produce electrostatic forces of 20 nN between the tip and the PM phase. Such a large voltage is sufficient to cause resistive switching of LSMO.^[^ ^68^ ^]^ Furthermore, conducting AFM measurements of the *x =* 0.3 manganite film in both low and high‐temperature phases (see Figure S14, Supporting Information) show that currents of 100 nA would be generated by a bias voltage of 6 V. This would lead to a large current density of 2 MA cm^−2^ at the contact, easily sufficient to produce large temperature rises. The behavior shown in Figure 1 allows us to rule this out. We thus find no support for the idea that electrostatic forces due to contact potential differences, contact electrification, or tribocharging can explain the observed increase in friction at the phase transition.

### 2.5 Excess Friction in the Small Polaron Hopping Conductivity State

The clear increase in friction at the transition from the metallic to polaronic state (Figures 1 and 2) in both the *x =* 0.2 and *x =* 0.3 films shows that the excess friction results from the properties of the polaronic state. The absence of a corresponding increase in adhesion at the transition reveals that contributions from changes in the interatomic bonding or in the contact at the transition are insignificant, so that the excess friction must result directly from changes in the inherent damping of the near surface region of the LSMO. Further, the thermally activated behavior and the logarithmic scan velocity dependence of the friction coefficient point to control by stick‐slip events in both the metallic and polaronic conducting states (Figure 3). Thus, the interaction of the tip with the sample generates the same stick‐slip pulse excitations above and below the transition, but these slip pulses are damped more strongly in the PM phase due to their interactions with small polarons. The idea of adding a viscous dissipation channel due to interactions of the tip with electronic degrees of freedom, as has been considered in previous publications (e.g., *F*_f_ *= F*_ph_+*F*_el_ = (*μ*_ph_+*μ*_el_)*F*_N_)^[^ ^7^, ^38^, ^40^, ^41^, ^43^ ^]^ is not relevant here. Furthermore, as has been noted previously, estimates of the magnitude of possible electronic contributions *F*_el_, whether due to van der Waals interactions or to Ohmic losses, are several orders of magnitude too small to explain the observed changes in sliding friction both in the literature^[^ ^7^, ^38^, ^40^, ^41^, ^43^ ^]^ and in our study.

**Figure 5:**
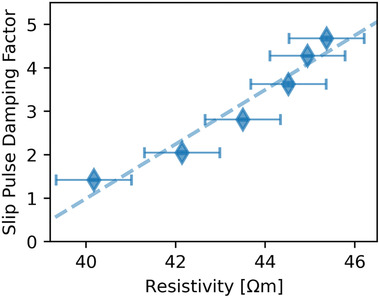
The slip pulse damping factor in the *x =* 0.3 film polaronic state scales with the resistivity.

We suggest instead that the friction force can be expressed as *F*_f_ *= α*_P_ *F*_M_(*T*) where *F*_M_(*T*) is the friction force needed to generate slip pulse excitations in the metallic state (e.g., the dashed line in Figure 2b) and *α*_P_ is a slip pulse‐polaron damping factor that reflects the strength of the slip pulse coupling to small polarons. The polarons that emerge around the transition temperature in LSMO consist of *e*_g_ electrons localized on dynamic Jahn–Teller distorted Mn^3+^.^[^ ^47^, ^48^, ^49^, ^50^, ^51^ ^]^ This transition is also reflected in the electrical resistivity (Figures S4 and S9, Supporting Information), which is governed by the double‐exchange mechanism well below the transition, but increases on passing through the transition as the Jahn–Teller energy increases and electron‐phonon coupling becomes stronger.^[^ ^47^, ^48^, ^49^, ^50^, ^51^ ^]^ The resistivity increases further with temperature until the electron‐phonon coupling is strong enough that hopping polaronic conduction sets in with an accompanying decrease in resistivity (Figures S4 and S9, Supporting Information).^[^ ^48^, ^49^, ^50^, ^51^ ^]^ In fact, the slip pulse‐polaron damping factor defined above for the *x =* 0.3 film scales very well with the electrical resistivity in the neighborhood of the transition temperature where small polarons begin to form (**Figure** **5**). This provides support for the idea that the vibrational slip‐pulse excitations couple strongly to the phonon degrees of freedom, and through them, to the electron degrees of freedom as a result of the strong electron‐phonon coupling which emerges around the transition. Coupling to the magnetic degrees of freedom will also occur, but is expected to be weaker than the electron‐phonon coupling effects in the temperature range investigated here;^[^ ^22^ ^]^ furthermore, there is no evidence of an effect of the FM transition in our studies (Figure 2b).

We are now faced with the question of whether the coupling of slip‐pulses to polarons can account for an increase by a factor of 5 in the slip pulse damping *α*_P_ around the transition temperature (Figure 2b). The dominant phononic contribution to damping at the low velocities used for studying friction comes from the generation of sound waves,^[^ ^1^, ^18^, ^69^, ^70^, ^71^ ^]^ as considered for the dissipation of adsorbate vibrations.^[^ ^1^, ^72^ ^]^ It is argued that the rate of frictional energy dissipation is proportional to *ω* ^2^ or *ω* ^4^ depending on whether the phonons with mode frequency *ω* are laterally coupled or oscillate independently, respectively.^[^ ^36^ ^]^ In the case of *x =* 0.3 material, a large change in the phonon spectrum is documented at the transition,^[^ ^73^, ^74^ ^]^ with the appearance of high wavenumber peaks associated with Jahn–Teller bond‐stretching^[^ ^74^ ^]^ above the transition temperature. Assuming a complete shift of the populated phonon states from Mn—O bond excitations at ℏ*ω* ≈ 47 meV^[^ ^76^ ^]^ below the transition temperature to the Jahn–Teller peaks at ℏ*ω* ≈ 62 meV and ℏ*ω* ≈ 78 meV above the transition,^[^ ^72^, ^75^ ^]^ would result in an increase in friction by factors between 1.8 and 7.5, which could account for the observed friction increase by a factor of almost 5. It remains to argue that the slip pulses couple strongly to the Jahn–Teller modes. This will depend on complex details of the slip pulse excitations and the interatomic potentials. One possible reason for a strong coupling will be discussed in the following section. Another is based on the fact that slip pulses at the surface of LSMO are not only vibrational excitations; due to the different interatomic bonding and valence states in LSMO, slip pulse motion will induce polarization and lead to dielectric excitations of the Mn—O octahedra and thereby to the Jahn–Teller modes.

### 2.6 Slip Pulse Model for Friction with Explicit Damping Dependence

Given the success of the Prandtl–Tomlinson model in accounting for essential features of friction behavior, it is appealing to consider whether it can be extended to explicitly include damping of the slip pulse excitations. As already mentioned, most studies have focused on the critically‐damped regime of spring‐driven Prandtl–Tomlinson models where there is no explicit dependence of friction on damping.^[^ ^27^ ^]^ Critically‐damped behavior is supported by atomic stick‐slip experiments which rarely show evidence of barrier re‐crossing (overdamping) or inertial multi‐slip (underdamping).^[^ ^27^ ^]^ Furthermore, a recent simulation study^[^ ^35^ ^]^ argues that phonon “dephasing”^[^ ^73^ ^]^ in reaction to an atomic stick‐slip event will lead to emergent critically‐damped behavior. Which damping regime is relevant depends on the ratio of the inherent dissipation rate in the material *η* to the local undamped contact resonance frequency $\sqrt{k/m}$, where *k* is the contact stiffness and *m* is an effective mass of the asperity. Critical damping occurs when the two rates are approximately equal, while underdamping and overdamping occur for smaller and larger inherent dissipation rates, respectively. A careful numerical study of the effect of damping on thermally activated stick‐slip behavior shows that the friction force increases steeply with the dissipation rate in both the underdamped and overdamped regimes, and is relatively constant in the critically‐damped regime.^[^ ^29^ ^]^ Since local contact stiffnesses and effective masses depend on local atomic structure and normal stresses, all three damping behaviors may be simultaneously manifested in a general, incommensurate, nanoscale contact and an explicit dependence on the dissipation rate may be expected.

In this paper, we take a heuristic approach using guidance from the theory of dislocation glide to develop a model for nanoscale friction which explicitly includes inherent material dissipation.^[^ ^18^, ^19^ ^]^ In comparison with previously discussed scenarios, we take advantage of the 2D nature of the contact interface and the fact that the dynamic atomic‐level processes for slip pulse motion may be different, parallel, and perpendicular to the slip direction, just as the motion of dislocation is governed by overcoming the Peierls barrier to form a kink pair and then by the lateral motion of the kinks. The equation we obtain provides a framework to explain our observations, but we have not attempted a fully quantitative approach and do not claim the model is unique in being able to motivate our findings. However, the equation is successful in explaining the observed dependence of friction on the phase transition as well as on the temperature and scan velocity. Effectively, our equation describes underdamped Prandtl–Tomlinson behavior where the size of the slip step is controlled by viscously damped kink motion.

The well‐studied example of dislocation glide in crystals provides a helpful analogy for understanding the atomic mechanisms and role of damping in the motion of a slip pulse. The essential feature is that dislocation glide is controlled by two distinct mechanisms: initiation of glide by kink pair nucleation, followed by lateral motion of the kinks (perpendicular to the slip direction) apart from each other. Even if it is clear that the topological constraints on dislocation structure, glide, and kink motion do not apply to slip pulses in an incommensurate interface between two dissimilar materials, it is also clear that the 2D nature of the contact interface requires mechanisms for propagation both parallel and perpendicular to the slip direction. For a disordered interface with large local variations in slip barriers, one might expect that slip pulses may disappear after propagating only a short distance along and perpendicular to the slip direction. In the limit that these distances are atomic distances, the resultant atomic stick‐slip behavior provides the upper limit for the friction force at a fixed scan velocity.^[^ ^19^ ^]^ However, atomic stick‐slip is unlikely to be a major contributor to a general frictional contact, so that dislocation glide models based on non‐adiabatic creation of kink pairs followed by viscously damped lateral motion of the kinks, may capture the essentials of slip pulse motion at a sliding interface.

The unloaded activation energy barriers for slip at an incommensurate interface between two dissimilar materials that are not chemically bonded (*E*_o_ in Equation 4) are presumably quite a bit smaller than the Peierls barriers in single‐crystalline materials. Thus, given the large shear stresses acting on a slip pulse (here, *τ* ≈ 2 GPa), it is reasonable to assume that only forward‐moving kink pairs are generated in the sliding contact and that the large shear stress relative to the barrier height entirely hinders backward moving kink pairs. This is the same condition described by the critically‐damped 1D Prandtl–Tomlinson model for friction. Once a forward moving kink pair is nucleated, the large shear stresses in the interface will sweep the kinks laterally apart. Just as for dislocations, the barrier for lateral kink motion in a slip pulse is smaller than for kink nucleation. Therefore, we assume that kink velocity is controlled by drag, *v*_k_ *= τbw*_k_ *B*_k_^−1^, where *v*_k_ is the kink pair separation velocity, *τ* is the shear stress acting on the kinks (assumed for simplicity to be the same as that driving kink pair nucleation), *w*_k_ is the lateral distance swept out by the kink pair, and *B*_k_ *= η*_k_ *m* is a kink pair viscous damping coefficient that is dependent on *T*.^[^ ^76^, ^77^ ^]^ The kink pair dissipation rate *η*_k_ is the inverse of a kink pair lifetime. This then allows us to express the scan velocity as the product of the rate of kink pair generation in the contact *Aρ* exp(−Δ*E/kT))*, the kink pair velocity *v*_k_, and a factor *α’* describing the contribution of each kink pair to forward sliding,^[^ ^76^, ^78^ ^]^

(5) $$v=\alpha^{\prime }\cdot v_{k}\cdot A\rho \;\exp\left(\frac{-E_{o}+\frac{\Omega_{\mathrm{kp}}}{\pi A}F_{f}}{kT}\right)=\frac{\beta F_{f}}{B_{k}}\;\exp\left(\frac{-E_{o}+\frac{\Omega_{\mathrm{kp}}}{\pi A}F_{f}}{kT}\right)$$

where we have used Equation (1) to Equation (3) and replaced *b* ^2^ *w* in the 1D expression for Δ*E* (Equation 4) with the volume *Ω*_kp_ of the kink pair nucleus. Furthermore, we have defined *β = α’bw*_k_ *ρ*, which is a geometrical measure of the contribution of all kink pairs to forward sliding.^[^ ^77^ ^]^ This equation has a similar form to Equation (3), but with the essential difference that the scan velocity pre‐factor has now been replaced with a term proportional to the friction force and inversely proportional to a damping coefficient in the material. Just as for the Prandtl–Tomlinson model, this equation describes the generation of stick‐slip events by non‐adiabatic thermal activation of kink pairs, but in this case, the contribution of each slip pulse to the scan velocity is determined by the viscously damped motion of the kink pairs in the interface.

Equation (5) gives fits to the scan velocity dependence of the friction force (Figure 3) that are indistinguishable from those of Equation (4), but the interpretation of the fitting parameters is different. In contrast to the temperature dependence of *b* obtained from fitting with Equation (4), the value of *Ω*_kp_ from the fit with Equation (5) is roughly temperature independent, which seems physically reasonable. Meanwhile, *b* increases by a factor of around 4 as the temperature increases from 112 to 232 K, consistent with the idea that the damping increases in the polaronic phase by the same factor as the friction. Except at low temperatures, the dominant dislocation drag effects are due to the phonon subsystem which generally behaves viscously.^[^ ^18^, ^77^ ^]^ The largest phonon loss contribution at low dislocation glide velocities is due to the generation of sound waves (phonon radiation friction), and has been estimated for the case of kinks moving along straight dislocations.^[^ ^78^ ^]^ According to phonon coupling, a kink of width *D* will couple most strongly into phonons with wavelengths ⪆2*D*.^[^ ^78^ ^]^ Since the barrier to kink motion is assumed to be small, *D* will be larger than the atomic spacing, so that the main coupling to the Jahn–Teller modes of the Mn—O octahedral occurs via an electron‐phonon coupling.

We note that in addition to the explicit appearance of a viscous damping coefficient in Equation (5), there is also a dependence on the friction force *F*_f_ in the pre‐factor. This gives the velocity equation features of both strongly driven systems (seen as a reduction of activation barrier by the friction force) and a weakly driven system (linear dependence on the driving force). Both the linear and exponential dependence of the scan velocity *v* on the friction force *F*_f_ result in a range of possible behaviors. At constant temperature, the scan velocity is more sensitive to changes in the friction force through the exponential term than through the linear term, explaining the observed logarithmic dependence of the friction force on scan velocity (Figure 3).

## 3 Conclusion

We report a clear increase in friction of two different LSMO films that correlates with the formation of Jahn–Teller distortions and small polarons. We are able to reproduce the temperature and scan velocity dependence of the friction and provide an order of magnitude justification based on the picture that the slip pulses generated in the sliding interface are damped by coupling to the phonon bath. In the case of the LSMO, this coupling increases at the transition temperature due to the emergence of small polarons in the near‐surface region of the material. We believe this model, which combines the widely accepted stick‐slip behavior at sliding contacts with concepts for damping of moving dislocations, provides a solid basis for interpreting nanoscale friction, also in other materials. We emphasize that our interpretation of the frictional dissipation mechanism is distinct from other studies where viscous electronic damping of the tip motion has been discussed but also recognized as much too small to account for the observed effects. Future studies may take advantage of tailoring phonon degrees of freedom in materials to better understand the damping of slip pulses as well as to develop tactics to control friction. In this respect, 2D layered materials are particularly promising, since they offer a number of tactics for phonon engineering.^[^ ^80^ ^]^

## 4 Experimental Section

##### LSMO Films

The *x =* 0.3 film (La_0.7_Sr_0.3_MnO_3_) was deposited on a (001) SrTiO_3_ substrate with a La_0.4_Sr_0.6_MnO_3_ buffer layer using the metal‐aerosol deposition technique.^[^ ^81^ ^]^ *Θ–*2*Θ* X‐ray diffraction experiments (XRD) showed no indications of any impurity phase and confirmed highly oriented growth on the substrate (Figure S1, Supporting Information). Small‐angle X‐ray scattering gave thicknesses of 5.8(2) nm and 5.4(2) nm for the *x*  =  0.3 film and buffer layer, respectively (Figure S2, Supporting Information). The *x*  =  0.3 film undergoes the typical second‐order phase transition from an FM metal to PM metal which was accompanied by a change in the sheet film resistance with increasing temperature, which is characteristic for the formation of Jahn–Teller polarons.^[^ ^46^, ^47^, ^48^, ^49^, ^50^ ^]^ The transition was characterized using SQUID magnetometry (Figure S3, Supporting Information) and four‐point sheet resistance measurements (Figure S4, Supporting Information). The metal‐metal transition temperature extracted from the resistivity data was *T*_MM_ = max(*ρ* ^−1^d*ρ*/d*T*) = 330 K and the Curie temperature extracted from the magnetometry data was *T*_C_ = max(d*μ*/d*T*) = 338 K. According to the phase diagram,^[^ ^82^ ^]^ this transition occurred in the 5.8 nm thick La_0.7_Sr_0.3_MnO_3_ layer, while the buried La_0.4_Sr_0.6_MnO_3_ buffer layer remained a PM‐metal above room temperature and at all temperatures probed in this study. The specimen has an RMS‐roughness of approximately 0.7 nm (Figure S5, Supporting Information), extracted from 500 × 500 nm^2^ AFM topography scans, which did not change as the temperature was cycled.

The *x*  =  0.2 film (La_0.8_Sr_0.2_MnO_3_) was deposited on a (001) SrTiO_3_ substrate by sputter deposition.^[^ ^52^ ^]^ *Θ*–2*Θ* XRD showed no indications of any impurity phases and confirmed highly oriented growth on the substrate (Figure S6, Supporting Information). The film thickness of 70 nm was determined by small‐angle X‐ray scattering (Figure S7, Supporting Information). The film undergoes a phase transition from an FM metal to PM metal which was characterized using SQUID magnetometry giving a Curie temperature at *T*_C_ = max(d*μ*/d*T*) = 222 K (Figure S8, Supporting Information). The resistance measurement associated with the phase transition was characterized using a temperature‐dependent four‐point measurement (Figure S9, Supporting Information). This yielded a transition from the metal to polaronic state at *T*_MM_ = max(*ρ* ^−1^d*ρ*/d*T*) = 186.8 K. By tuning the deposition temperature and optimizing deposition parameters, the transition temperature was reduced by more than 100 K compared to the phase diagram,^[^ ^82^ ^]^ presumably due to stresses from the epitaxial relation with the substrate and from sputter preparation‐induced point defects.^[^ ^52^ ^]^ The film showed an RMS‐roughness of less than 0.2 nm, extracted from 500 × 500 nm^2^ AFM topography scans (Figure S10, Supporting Information).

##### AFM Measurements

AFM experiments were performed with a commercial Omicron VT‐AFM/STM in a vacuum chamber at a base pressure of *p =* 10^−10^ mbar. The La_0.7_Sr_0.3_MnO_3_ film (*x =* 0.3) was radiatively heated from the back and the sample surface temperature was directly calibrated before experiments were performed. For measurements on La_0.8_Sr_0.2_MnO_3_ (*x =* 0.2), the sample was clamped to a cryogenic stage whose temperature was controlled by adjusting the heating power of a resistor integrated into the stage and the liquid nitrogen flow. The surface temperature of the specimen was expected to be around 20 K warmer than the stage due to the poor thermal contact between the stage and the lightly clamped specimen.

Commercially available rectangular, single crystalline silicon cantilevers (Nanosensors PPP CONTSCR) with a nominal tip radius of less than 10 nm were used (although a scanning electron microscopy (SEM) image, Figure S11, Supporting Information, suggests an actual radius of 21 nm). The normal *k*_n_ and torsional *k*_t_ cantilever spring constants used for the *x =* 0.3 measurements were obtained from the manufacturer values for the median cantilever dimensions and literature values for elastic constants (*k*_n_ *=* 0.52 N m^−1^ and *k*_t_ *=* 42.74 N m^−1^), while the normal and lateral forces were calibrated using the procedures described in the literature.^[^ ^82^, ^83^, ^84^, ^85^ ^]^ The La_0.8_Sr_0.2_MnO_3_ specimen (*x =* 0.2) was measured with an un‐calibrated, pre‐mounted cantilever of the same type as for the *x =* 0.3 film. Estimates of the spring constants were obtained by assuming the average pull‐off forces are the same for the *x =* 0.2 and *x =* 0.3 specimens (Figure 1e), leading to normal spring constants of *k*_n_ *=* 0.03 and 0.02 N m^−1^ for the first and second *x =* 0.2 measurements, respectively. These values are lower than for the *x =* 0.3 cantilevers, presumably due to variations in cantilever dimensions but are still within the manufacturer specifications. Assuming they result from a decreased cantilever thickness, values for the torsional spring constants were obtained for the cantilevers used in the first and second measurements of the *x =* 0.2 film of *k*_t_ = 15.02 N m^−1^ and *k*_t_ = 9.87 N m^−1^, respectively.

Nanoscale friction measurements were performed using AFM‐based lateral force microscopy.^[^ ^56^ ^]^ The lateral forces during sliding at a constant scan velocity and under a constant applied normal force were obtained from the measured torsion of the cantilever. By measuring the torsion during friction loops (trace and retrace scanning along the same line on the film surface, Figure 1a), the effects of topography were largely separated out from friction effects.^[^ ^56^ ^]^ The friction forces *F*_f_ were obtained by averaging 100 or 256 friction loops that were performed in 100 × 100 nm^2^ or 500 × 500 nm^2^ regions on the surface of the specimens for each normal load and temperature of interest. To minimize wear during the friction experiments, the applied normal forces *F*_N_ were kept below 30 nN and any possible changes in the contact were monitored through tip‐sample adhesion measurements. Adhesion forces were obtained by recording 100 force‐distance curves before and after probing the frictional properties at each temperature and determined by averaging the extracted pull‐off forces.

##### Statistical Analysis

The friction and adhesion data were obtained in three independent sets of experiments on two different LSMO thin film samples. To eliminate contributions from the specific film deposition method, two different deposition methods were used: sputter deposition for the *x* = 0.2 film and metal‐aerosol deposition^[^ ^81^ ^]^ for the *x* = 0.3 film. The large difference in the transition temperatures of the two films allowed experiments to be performed in very different temperature ranges and to rule out possible thermal artifacts. To rule out effects from tip wear, the first and last measurements in a given series were performed at the same temperature.

The data evaluation was performed with user‐written Python and MATLAB scripts. Each friction force or adhesion force data point represents the mean value of 100 (*x* = 0.2 film) or 256 (*x* = 0.3 film) individual friction loops or pull‐off measurements, while the displayed error is the standard error.

Correlations between the friction forces and surface height, height gradient, and surface curvature were ruled out on the basis of the Spearman *r*_S_ coefficients (−0.11 ≤ *r*_S_ ≤ 0.1) (see Figures S12 and S13, Supporting Information).

## Conflict of Interest

The authors declare no conflict of interest.

## Supporting information

Supporting Information
