# Supplementary material for: Polaronic Contributions to Friction in a Manganite Thin Film
Source: Adv Sci (Weinh). 2021 Feb 18;8(8):2003524. doi: 10.1002/advs.202003524 (PMC8061368; doi:10.1002/advs.202003524)
Supplement: Supplementary file 1 — Supporting Information [file ADVS-8-2003524-s001.pdf]

WILEY-VCH

© 2020 Wiley-VCH GmbH

ADVANCED SCIENCE
Open Access

Supporting Information

for *Adv. Sci.*, DOI: 10.1002/advs.202003524

Polaronic Contributions to Friction in a Manganite Thin Film

*Niklas A. Weber, Dr. Hendrik Schmidt, Tim Sievert, Prof. Christian Jooss, Dr. Friedrich Güthoff, Prof. Vasily Moshneaga, Prof. Konrad Samwer, Prof. Matthias Krüger, and Prof. Cynthia A. Volkert**

Supporting Information

Polaronic Contributions to Friction in a Manganite Thin Film

Niklas A. Weber[1], Dr. Hendrik Schmidt[1], Tim Sievert[1], Prof. Christian Jooss[1,5], Dr. Friedrich Güthoff[2], Prof. Vasily Moshneaga[3], Prof. Konrad Samwer[3,5], Prof. Matthias Krüger[4], and Prof. Cynthia A. Volkert[1,]*

[1] *Institute of Materials Physics, University of Göttingen, 37077 Göttingen, Germany*

[2] *Institute of Physical Chemistry, University of Göttingen, 37077 Göttingen, Germany*

[3] *1. Physics Institute, University of Göttingen, 37077 Göttingen, Germany*

[4] *Institute for Theoretical Physics, University of Göttingen, 37077 Göttingen, Germany*

[5] *The International Center for Advanced Studies of Energy Conversion (ICASEC), University of Göttingen, 37077 Göttingen, Germany.*

E-mail: volkert@ump.gwdg.de

Section S1. Sample Characterization

The $La_{0.7}Sr_{0.3}MnO_3$ film deposited by metal-aerosol vapor deposition (**Figure S1-S5**) and the $La_{0.8}Sr_{0.2}MnO_3$ desposited by sputter deposition (**Figure S6-S10**) were characterized with several methods. θ-2θ x-ray diffraction experiments (XRD) and x-ray reflectometry (XRR) were carried out using a Bruker D8 with Cu-K_α source (**Figure S1, S2, S6, and S7**). The XRD spectra show (001) family LSMO reflections superimposed on strong reflections from the (001) oriented single crystal $SrTiO_3$ substrate (**Figure S1 and S6**), confirming the heteroepitaxial relation between the film and substrate. Diffration peaks from the aluminum

sample holder peaks can also be seen. The XRR spectra (**Figure S2 and S7**) were fit to obtain the thicknesses of the deposited LSMO films.[S1]

The magnetic and electrical properties of the specimens were measured using SQUID (superconducting quantum interference device) and four-point resistivity (**Figure S3, S4, S8, and S9**) and the transition temperatures determined using the relations $T_C = max(d\mu/dT)$ and $T_{MM}=max(\rho^{-1}d\rho/dT)$.

Topography maps (**Figure S5(a) and S10(a)**) were obtained using standard contact AFM methods with a commercial Omicron VT-AFM/STM in a vacuum chamber at a base pressure of p = 10^{-10} mbar.

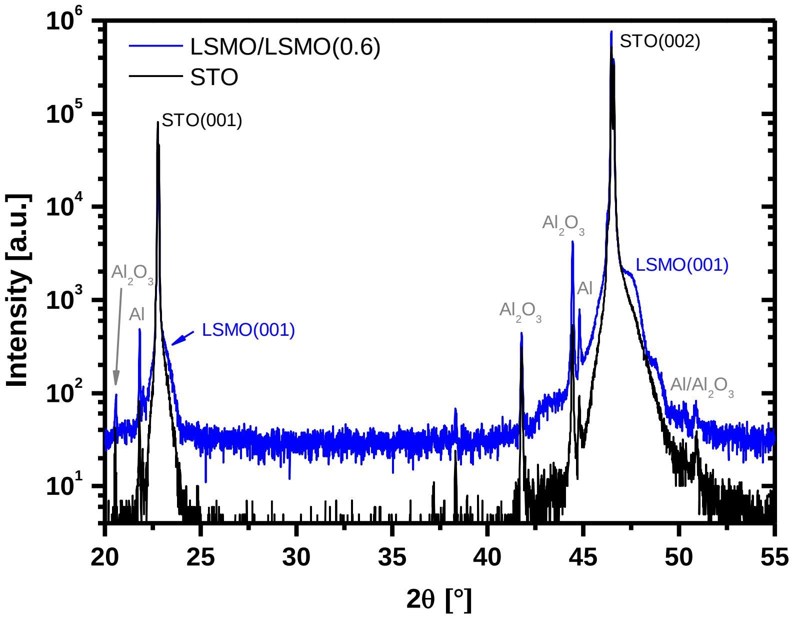

Figure S1. θ-2θ XRD measurements of the $La_{0.7}Sr_{0.3}MnO_3/La_{0.4}Sr_{0.6}MnO_3$ bilayer which was deposited on a (001) oriented $SrTiO_3$ substrate. The lattice constants were determined to be 3.871(3) Å und 3.781(2) Å, respectively.[S2]

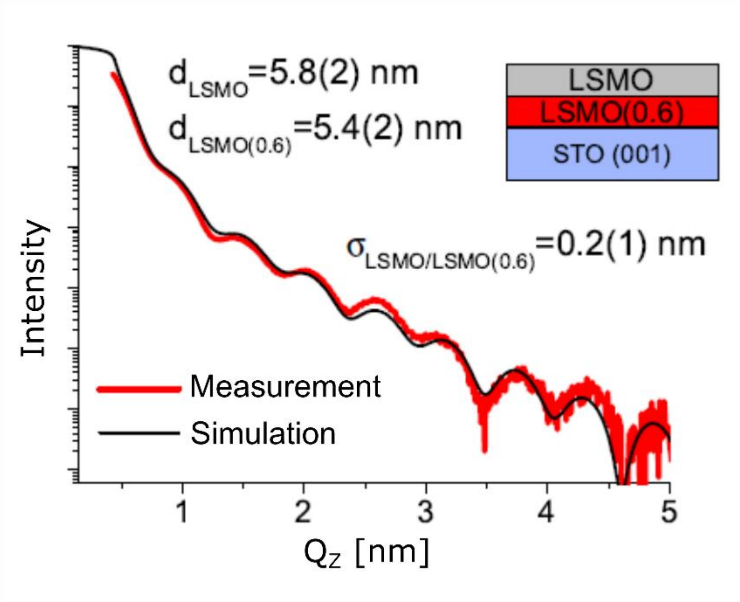

Figure S2. XRR measurements of the $La_{0.7}Sr_{0.3}MnO_3$/$La_{0.4}Sr_{0.6}MnO_3$ bilayer film yield thicknesses of 5.8 and 5.4 nm, respectively.[S2]

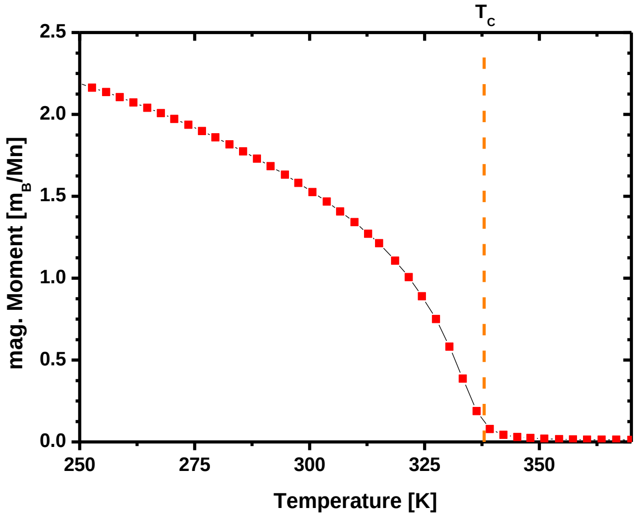

Figure S3. SQUID magnetometry measurements carried out on $La_{0.7}Sr_{0.3}MnO_3$/$La_{0.4}Sr_{0.6}MnO_3$ yield a Curie Temperature of T_C = 338 K.

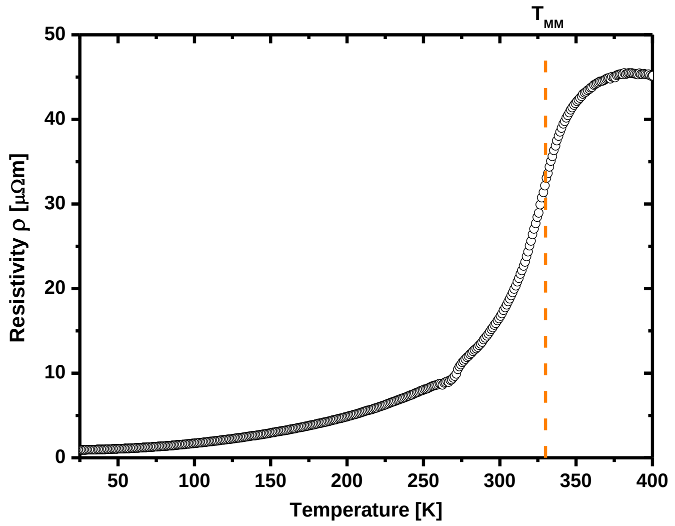

Figure S4. $La_{0.7}Sr_{0.3}MnO_3$/$La_{0.4}Sr_{0.6}MnO_3$ film resistance measured in a four-point geometry. The metal-metal transition temperature is found at T_{MM} = 330 K.

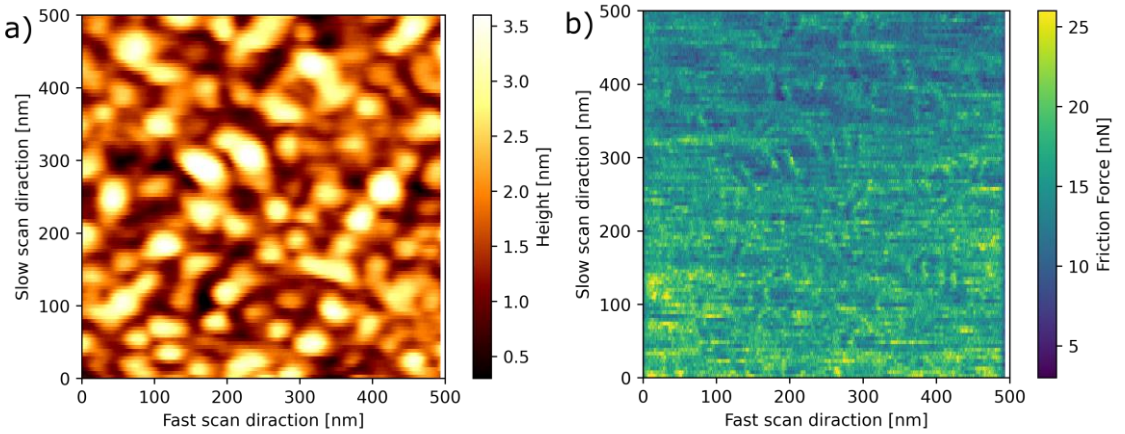

Figure S5. $La_{0.7}Sr_{0.3}MnO_3$ film (a) topography and (b) corresponding friction map measured at room temperature at a normal force of $F_N = 7.5$ nN under UHV conditions using an Omicron VT-AFM/STM. The specimen shows an RMS-roughness of approximately 0.7 nm. No effect of temperature on surface morphology was observed (not shown).

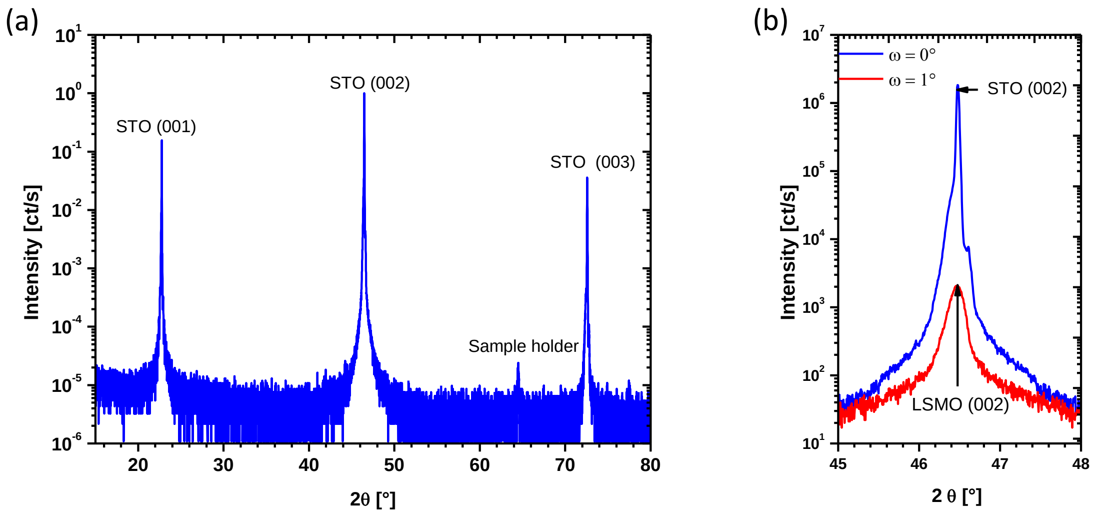

Figure S6. (a) θ-2θ XRD measurements of $La_{0.8}Sr_{0.2}MnO_3$ deposited on a (001) oriented $SrTiO_3$ substrate. The peaks from the $La_{0.8}Sr_{0.2}MnO_3$ are superimposed on the STO (001) peaks. (b) To distinguish substrate from film a second measurement for 2θ between 45° and 50° at ω=0° and 1° were conducted. A lattice constant of 3.906(2) Å was determind from the spectra.

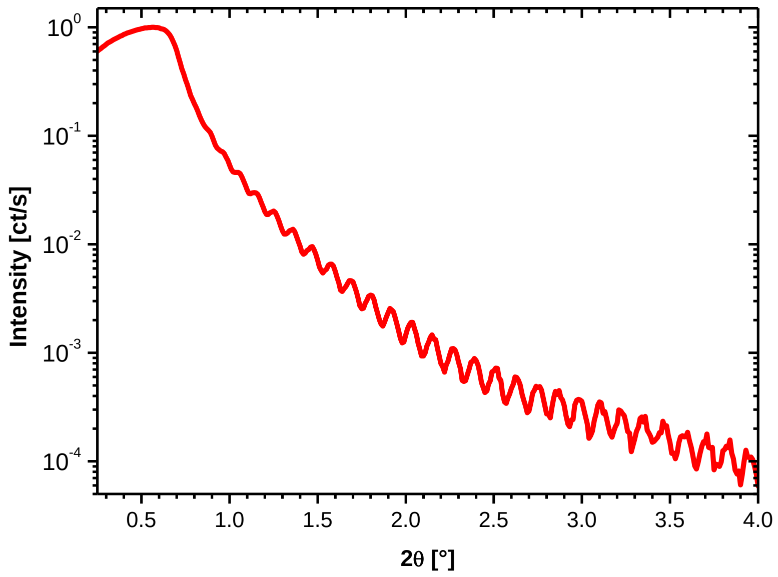

Figure S7. XRR measurements carried out on sputtered $La_{0.8}Sr_{0.2}MnO_3$ on $SrTiO_3$ yield a film thickness of 70 nm.

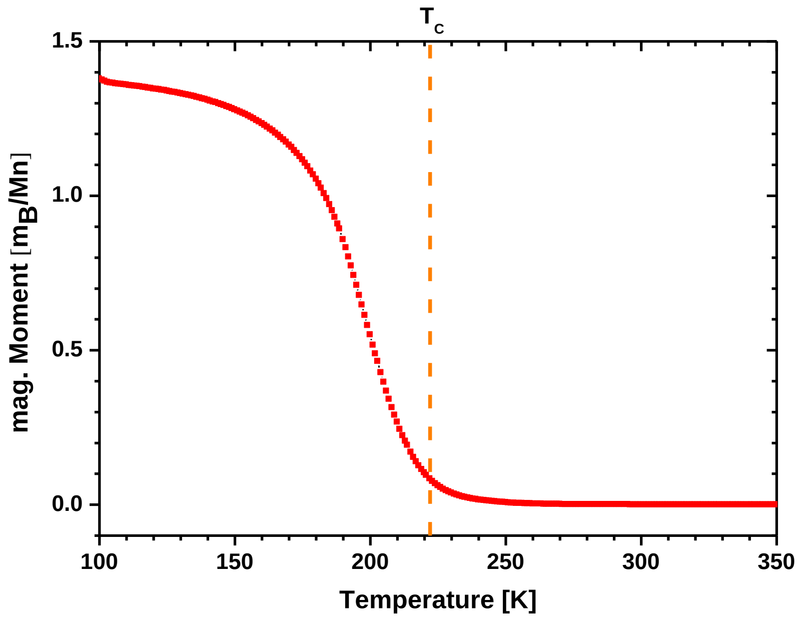

Figure S8. SQUID magnetometry measurements carried out on $La_{0.8}Sr_{0.2}MnO_3$ yield a Curie Temperature of $T_C = 222$ K.

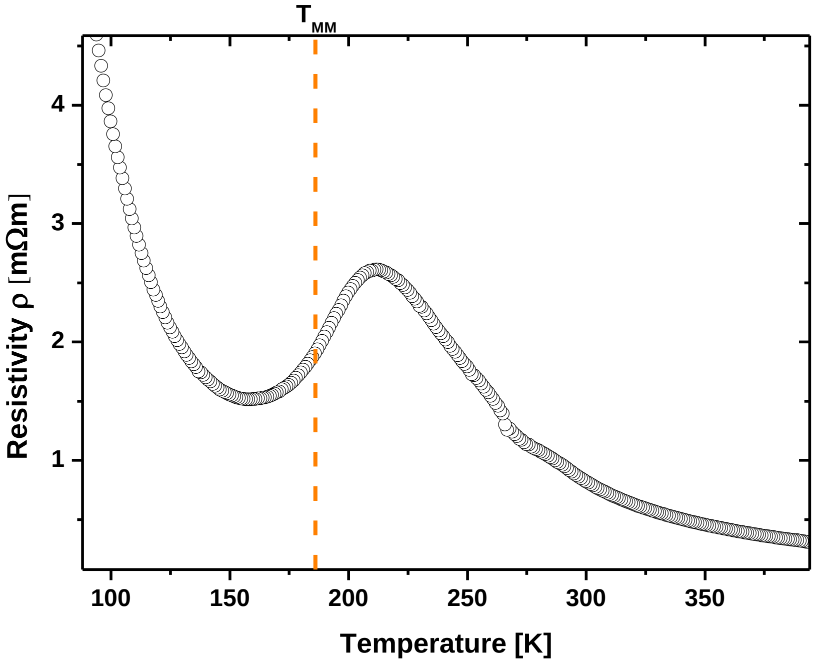

Figure S9. Four-point resistivity measurements on $La_{0.8}Sr_{0.2}MnO_3$ reveal a metal-metal transition temperature at T_{MM} = 187 K.

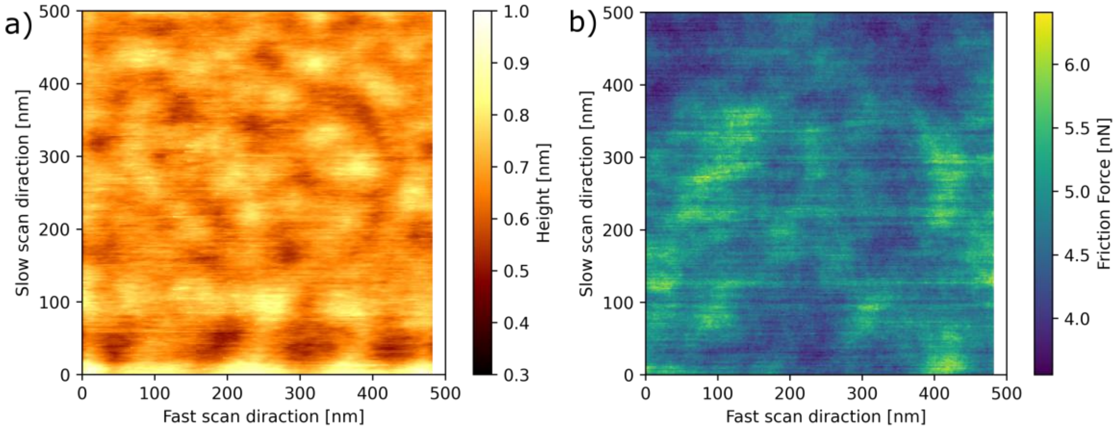

Figure S10. $La_{0.8}Sr_{0.2}MnO_3$ film (a) topography and (b) corresponding friction map measured at room temperature at a normal force of $F_N = 0.7$ nN. The RMS roughness remained constant below 0.2 nm during the entire temperature series friction measurements. No effect of temperature on surface morphology was observed (not shown).

Section S2. Cantilever Characterization

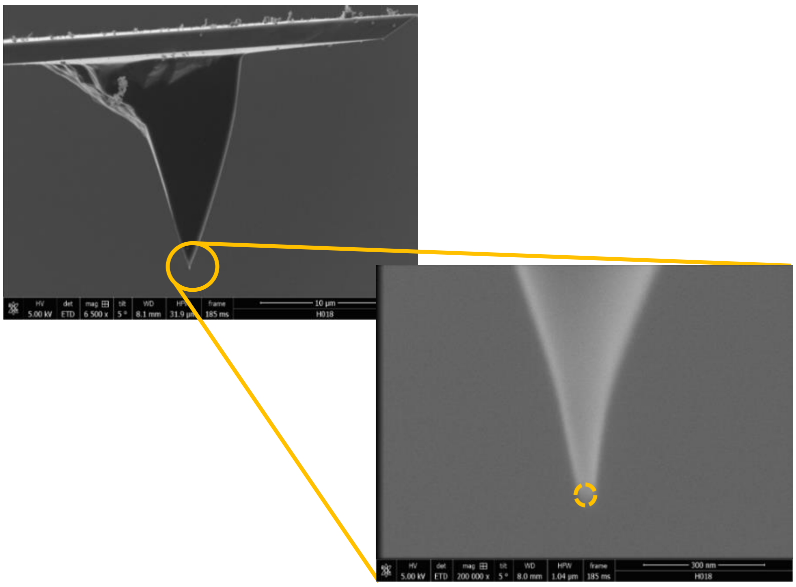

Figure S11. Scanning electron microscopy images from the tip of a cantilever (Nanosensors PPP-CONTSC) allowed an estimation of the tip radius as 21 nm.

Section S3. Effects of Topography on Friction

To test for any correlations between friction forces and surface height, height gradient, or surface curvature, Spearman correlation coefficients r_s were calculated. Representative examples are shown in **Figures S11 and S12** for the $La_{0.7}Sr_{0.3}MnO_3$ and $La_{0.8}Sr_{0.2}MnO_3$ films, respectively. The obvious lack of correlation is supported by the small correlation coefficient values and validates the lateral friction force measurement method.

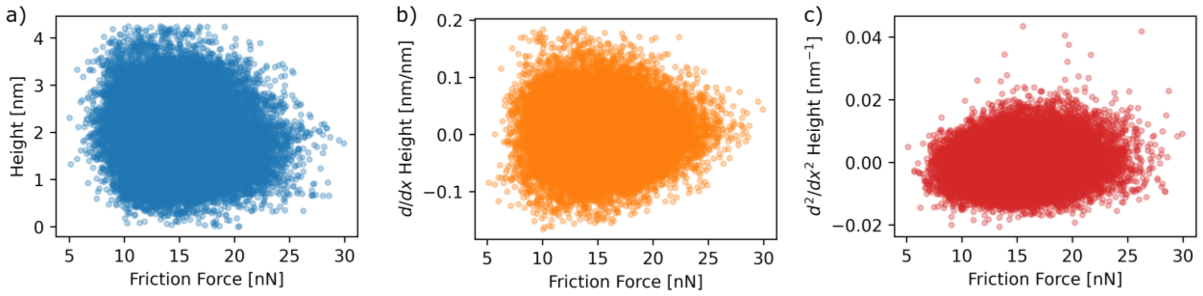

Figure S12. Scatter plots for the $La_{0.7}Sr_{0.3}MnO_3$ film of friction force vs (a) surface height r_s = -0.11, (b) height gradient r_s = -0.03, and (c) surface curvature r_s = 0.09 reveal little or no correlation between friction and topography.

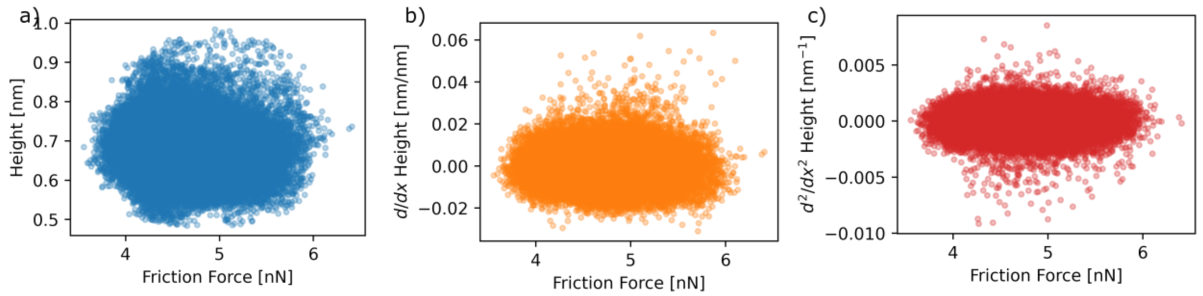

Figure S13. Scatter plots for the $La_{0.8}Sr_{0.2}MnO_3$ film of friction force vs (a) surface height r_s = -0.08, (b) height gradient r_s = -0.04, and (c) surface curvature r_s = 0.02 reveal little or no correlation between friction and topography.

Section S4. Electrical Characterization using C-AFM

Conductive-AFM (C-AFM) measurements were carried out under UHV conditions using Pt-coated silicon cantilevers (Nanosensors PPP-CONTSCPt) with similar spring constants to the Si cantilever used in the friction study to gain a deeper understanding of the nanoscale electrical properties of the $La_{0.7}Sr_{0.3}MnO_3$ film. In a first step, we recorded several I-V curves in the ferromagnetic state at room temperature and in the paramagnetic state at *T*= 380 K. As shown in **Figure** the sample becomes more insulating in the high temperature phase. We attribute the non-Ohmic behavior at low currents to the presence of an insulating, electrically dead surface layer, which is often reported for perovskite manganites.[S3]

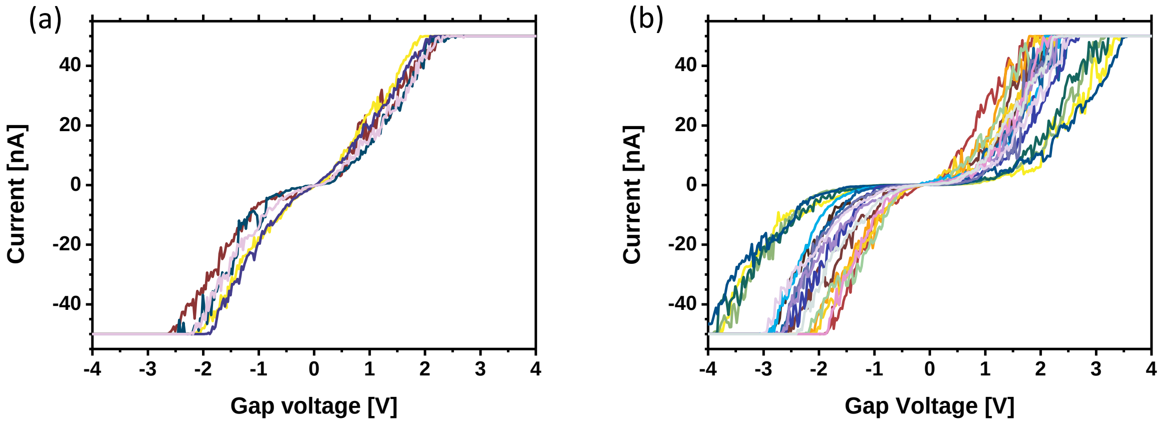

Figure S14. I-V spectroscopy curves for the $La_{0.7}Sr_{0.3}MnO_3$ film recorded at (a) room temperature where the sample is a ferromagnetic metal and (b) at T= 380 K where it is a paramagnetic metal. There is a clear increase in the resistance of the high temperature phase.

[S1] **References** L. Spieß, G. Teichert, R. Schwarzer, H. Behnken, C. Genzel, *Röntgendiffraktometrie für Materialwissenschaftler, Physiker und Chemiker*, Vieweg+Teubner Verlag, **2009**.

[S2] M. Jungbauer, *Doctoral Thesis*, Universität Göttingen, January, **2016**.

[S3] R.P. Borges, W. Guichard, J.G. Lunney, J.M.D. Coey, F. Ott, *J. Appl. Phys.* **2001**, 89(7), 3868-3873.
